# Physical Realizations of Interdependent Networks: Analogy to Percolation

**DOI:** 10.3390/e27020109

**Published:** 2025-01-23

**Authors:** Bnaya Gross, Shlomo Havlin

**Affiliations:** 1 Network Science Institute, Northeastern University, Boston, MA 02115, USA; 2 Department of Physics, Northeastern University, Boston, MA 02115, USA; 3 Department of Physics, Bar-Ilan University, Ramat-Gan 52900, Israel; havlins@gmail.com; 4 Tokyo Institute of Technology, Yokohama 226-8501, Japan

**Keywords:** interdependent networks, percolation, physical networks, critical phenomena

## Abstract

Percolation on interdependent networks generalizes the well-studied percolation model in a single network to multiple interacting systems, unveiling spontaneous cascading failures, abrupt collapses, and high vulnerability. The main novelty of interdependent networks has been the introduction of two types of links, connectivity within networks and the dependency between them. The interplay between these two types of interactions results in novel critical phenomena and phase transitions. This abstract percolation paradigm was recently applied to magnetic networks, as an experimentally testable method for interdependent superconducting networks as well as to other systems like k-core percolation and overloaded networks. Here, we will review these physical applications and provide insights into several potential directions for the field of physically interdependent networks.

## 1. Introduction

Network theory utilizes percolation theory to evaluate the resilience of a complex network under random failures [1,2]. In a standard percolation process, a fraction of 1−p of nodes (or edges) is randomly removed from the network, whereby, above a critical value, $1-p_{c}$ breaks the network into small clusters compared with the original network. The giant connected component (GCC) is defined as the largest connected component among the remaining clusters with size (number of nodes) of the order of the network size *N* (the number of nodes in the network). The GCC is considered the functional part of the network and is often measured by its relative size to the original network size, $P_{\infty }$, while all small clusters are considered non-functional [3,4,5]. It has been found that a critical percolation threshold at $p=p_{c}^{II}$ exists above which the GCC exists $P_{\infty }>0$ and the network still functions. We call the critical threshold $p_{c}^{II}$ since the transition is a continuous second-order transition. As *p* decreases, the GCC, which represents the order parameter, continuously decreases as well, and below $p_{c}^{II}$, the network is completely fragmented $P_{\infty }=0$. For Erdős–Rényi networks [3,6,7,8], the percolation threshold can be found analytically $p_{c}^{II}=1/\langle k\rangle$, where 〈k〉 is the average degree of the networks, whereas, for the site percolation of a 2D square lattice network, the threshold was only found numerically $p_{c}^{II}≃0.5927$ [1,9]. This continuous second-order phase transition (Figure 2a) has been widely studied, especially its critical phenomena, which unveil a unique universality class of percolation.

## 2. Percolation on Interdependent Networks

**Figure 1:**
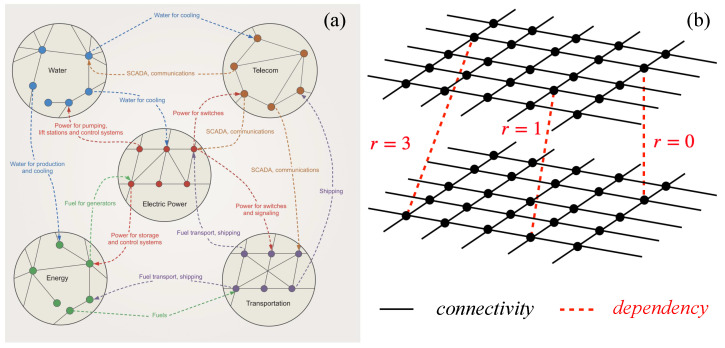
Interdependent networks. (**a**) Real-world interdependent networks of infrastructures. Figure obtained from Ref. [13]. (**b**) Two types of links exist: connectivity links (black solid lines) within networks for the function of each network and dependency links (dashed red lines) between the networks. In spatial networks, dependency links can be constrained to a limited spatial range *r* [14], where new types of transition occur, such as nucleation.

**Figure 2:**
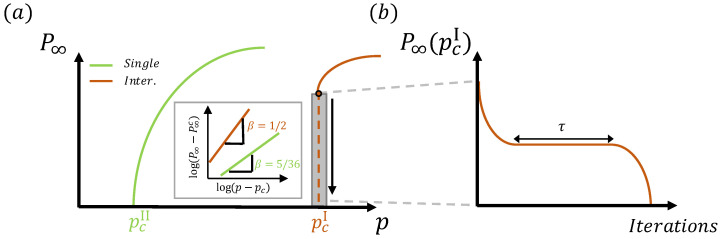
Percolation of interdependent networks. (**a**) In contrast to the second-order transition observed in percolation in a single network, interdependent networks are characterized by an abrupt transition. Inset: The critical exponent β=1/2 for interdependent networks and β=5/36 for a single 2D network (Equation (1)) characterize the scaling of the MGCC close to criticality. (**b**) At the critical point $p_{c}^{I}$, spontaneous cascading failures are observed in interdependent networks characterized by a *plateau* behavior of timescale τ, where the size of the MGCC remains almost constant for a long time. During this plateau, spontaneous microscopic changes occur. Along this plateau, a single node failure in one layer causes on average a single node failure in the other layer, at each step, with a branching factor of one.

The paradigm of interdependent networks was introduced when researchers realized that networks in nature and technology are usually not isolated [10]. Instead, these networks depend on each other to function properly (see Figure 1a). Interdependent networks are characterized by two types of links: *connectivity links* for forming the basic function of each network and *dependency links* form dependency relations between nodes in different networks (Figure 1b). When a percolation process is considered, dependency links between nodes propagate failures between the networks. If a node in one network fails, its dependent node in another network will fail as well. Since the GCC is considered as the functional part of the network after a percolation process, the interplay between the two types of interactions induces the appearance of a novel phenomenon of *cascading failures*. When a single node is removed, its dependent node in another network is removed as well, which disconnects some nodes from the GCC of their networks, resulting in their failure. The failure of these nodes could result in additional failures of their dependent nodes in the first network as well as in other networks, and so on. At the end of this process, the *mutual* giant connected component (MGCC) is measured (also annotated as $P_{\infty }$) and defined as the functional part of the entire system. Because of the dependency links between the networks at a certain *p* above $p_{c}^{II}$, a catastrophic cascade will spontaneously occur and the system will collapse. Below a critical percolation threshold, $p_{c}^{I}>p_{c}^{II}$, this cascading process results in an *abrupt transition*, unveiling the high vulnerability of interdependent networks (Figure 2a). This is in marked contrast to the continuous second-order transition observed in isolated networks. This abrupt transition was found to be of a mixed-order nature, having both abrupt jumps similar to first-order transitions and critical scaling as second-order transitions near $p_{c}^{I}$ [11,12]. A representative example of the critical scaling is the critical exponent β, characterizing the scaling of the order parameter close to the critical point (Figure 2a inset):

(1) $$P_{\infty }\left(p\right)-P_{\infty }\left(p_{c}^{I}\right)∼{\left(p-p_{c}^{I}\right)}^{\beta }.$$

The value β=1/2 was found to appear independently of the underlying topology of the networks, as long as the dependency links are long-range (r→L) [15]. Note that for a single network, Equation (1) is also valid but $P_{\infty }\left(p_{c}\right)=0$ (continuous transition) and β=5/36 (for d=2) [1,2,5]. Note also that the limited case of r=0 (for all nodes—see Figure 1) is identical to a single network. This is because for any damage made in one network, an identical damage occurs in the other network and there is no further cascading. Furthermore, it has been found that *during* the abrupt transition, a unique long-living *plateau* is observed (Figure 2b), where during the plateau, a *single* node failure in one network causes—on average in a single iteration step—the removal of a single node in another network. Thus, at criticality, at the plateau, the branching factor η is 1, analogous to the epidemic at criticality. The plateau time length, τ, that is, the number of iterations, was found to show critical behavior with the distance from the critical point $\tau ∼{\left(p-p_{c}^{I}\right)}^{-1/2}$ and also with the system size $\tau ∼N^{1/3}$ [16]. Other network models also experienced mixed-order transitions and similar cascading phenomena, including *k*-core percolation [17,18,19,20] and overload [21,22,23].

Despite the novel and rich phenomena observed in the theory of percolation in interdependent networks, an experimental proof for the theory was missing. There was no experimental setup for testing these theoretical predictions. At this stage, a path towards physical applications of interdependent networks was essential for proving its theoretical predictions. In the next two sections, we describe two physically coupled systems that have been found recently to have similar mixed-order transitions as in the percolation of interdependent networks. Those systems are interdependent ferromagnetic networks and interdependent superconducting networks. The last system was also explored experimentally while proving the paradigm of interdependent networks in real physical systems [24,25].

## 3. Interdependent Ferromagnetic Networks

While the first attempt to apply the paradigm of interdependent networks in physics was through interdependent resistor networks [26], it still remained under the percolation paradigm where dependency links between networks remain an abstract on–off relation. The main progress towards the physical manifestation of interdependent networks was in interdependent ferromagnetic networks (IFNs) [27,28]. An isolated ferromagnetic network is placed in a heat bath with the temperature T=1/β. Each node is an Ising spin $\sigma_{i}=\pm 1$, where the energy of each network is defined by the Hamiltonian of the Ising model [29], defining the energy of the system as the sum of the alignments of adjacent spins.

(2) $$H=-J\sum_{i<j}A_{ij}\sigma_{i}\sigma_{j}$$

where $A_{ij}$ is the adjacency matrix and *J* is the coupling strength. The order parameter of the system, which is analogous to the GCC in percolation, is the macroscopic magnetization, defined as

(3) $$M=\frac{1}{N}\sum_{i}\sigma_{i}.$$

At high temperatures, the system is at the disordered phase M≃0, while below a critical temperature, $T_{c}$ spontaneous magnetization appears, M>0, and the system experiences a continuous second-order phase transition similar to percolation in an isolated network. Nevertheless, the critical phenomena belong to a different universality class than percolation [1,30].

**Figure 3:**
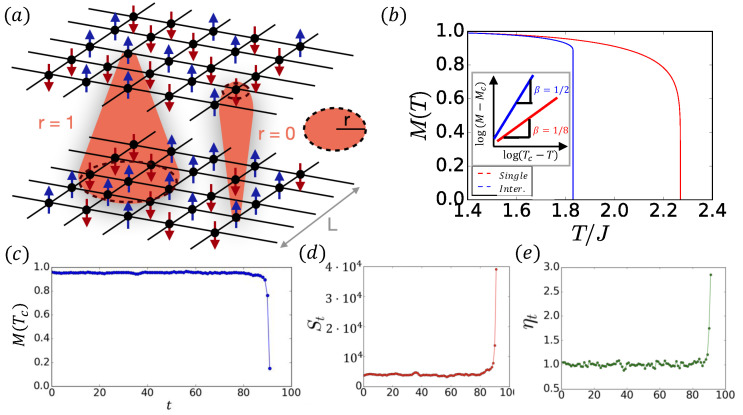
(**a**) Interdependent ferromagnetic networks. Two ferromagnetic networks are thermally coupled. Each node in a network is an Ising spin. Thermal coupling is a result of thermal dissipation associated with the local ordering of spins. The thermal range *r* affects the distance at which dissipation from one area of the network affects the other. To obtain a mixed-order transition, it is required that the range of the thermal coupling will be of the order of the system size. (**b**) In contrast to single ferromagnetic networks that experience a continuous second-order phase transition (red curve), interdependent ferromagnetic networks are characterized by an abrupt mixed-order transition. Inset: The critical exponents β=1/2 for interdependent ferromagnetic networks and β=1/8 for a single 2D ferromagnetic network. (**c**) During the abrupt transition, a plateau is observed with approximately constant magnetization before converging exponentially fast into the disordered phase. (**d**) During the plateau, the number of spins changing their state $S_{t}$ is approximately constant. (**e**) The constant value of changed spins yields a critical branching factor equal to 1, since $\eta_{t}=S_{t+1}/S_{t}≃1$. Figure obtained from Ref. [28].

IFN is composed of two ferromagnetic networks, *A* and *B*, coupled by thermal dissipation (Figure 3a). The thermal coupling is motivated by magnetoresistors [31,32]. Locally ordered spin results in a weak scattering of electrons and a low resistance, which creates weak thermal coupling due to weak dissipation. Disordered spins, on the other hand, create strong scattering, which results in high resistance and high thermal coupling. This thermal coupling is a physical manifestation of the abstract dependency links in percolation. The state of the spin depends on the local temperature, which is affected by the local order in the other network. This thermal coupling is reflected by the inverse temperature as

(4) $$\beta_{A,B}=\frac{M_{B,A}}{T}.$$

When the spins are ordered in one network (M≃1), the thermal coupling is very weak $\beta_{A,B}≃1/T$. However, when the system is not ordered (M≃0), a strong dissipation affects the temperature increase. This thermal coupling can create a thermal cascade between the networks. At a very low temperature, both networks are ordered (M≃1), and as the system is being heated, local disorder starts to appear. This disorder increases the temperature of the other network due to the thermal coupling (Equation (4)), which increases the disorder of the network. The increase in disorder in the network creates stronger heat dissipation that returns to the first network, and so on, until the entire system becomes completely disordered spontaneously (M≃0) in an abrupt transition (Figure 3b). Similar to percolation, in the long-range limit r→∞, a mixed-order transition is observed with the same critical exponent β=1/2 in Equation (1) [28] (Figure 3b inset). During the transition, a plateau is observed with approximately constant magnetization for a long time before converging to the disordered phase exponentially fast (Figure 3c). During the plateau, the average number of flipped spins, $S_{t}$, is approximately constant (Figure 3d), leading to a critical branching factor $\eta_{t}=S_{t+1}/S_{t}$ equal to one (Figure 3e). See also [25].

## 4. Interdependent Superconducting Networks

**Figure 4:**
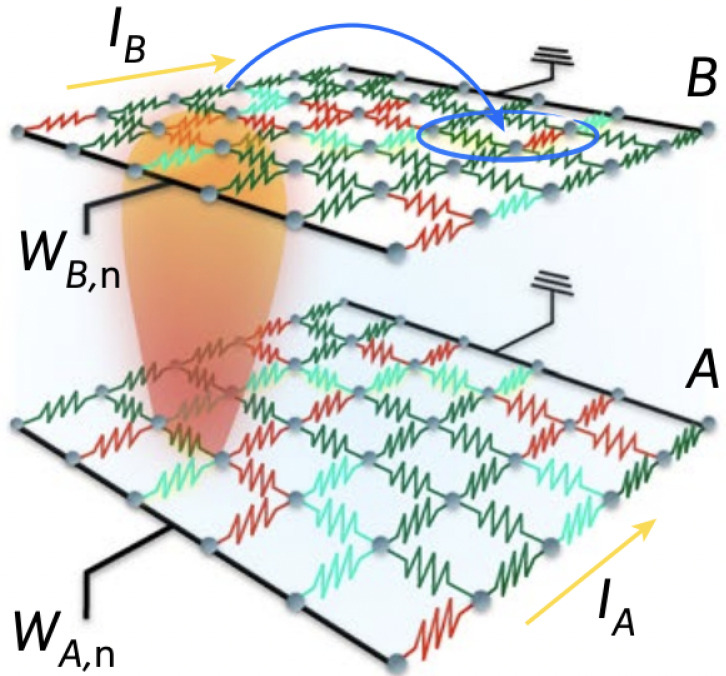
Interdependent superconducting networks. Two superconducting networks are thermally coupled. Each segment is characterized by Josesfon Junction characteristics. Interdependence is presented by thermal coupling, which is a result of thermal dissipation. Heat dissipation from one network to the other (or to the same) can change the state of a segment from SC-state to N-state, resulting in additional dissipation and a redistribution of current in the network, which results in cascading changes and an abrupt transition. Figure obtained from Ref. [24].

**Figure 5:**
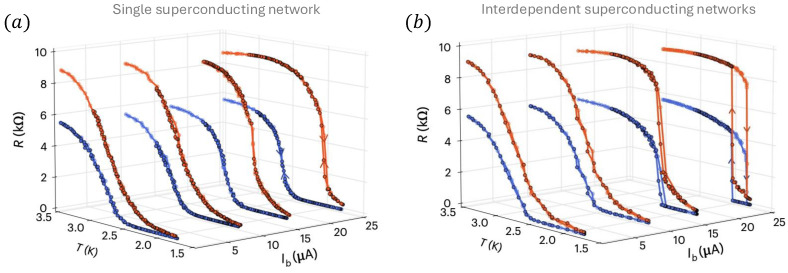
Phase transitions in single and interdependent superconducting networks’ experimental results. (**a**) A single superconducting network experiences a continuous second-order transition at a critical temperature $T_{c}$, which depends on the biased current $I_{b}$. This is analogous to the percolation of a single system (see Figure 2). (**b**) Similar to single networks, interdependent superconducting networks experience a second-order transition for low biased currents due to the weak thermal coupling. However, above a critical biased current $I_{b}^{c}$, the thermal coupling becomes strong enough to initiate a cascade and the transition becomes abrupt with hysteresis phenomena. Figure obtained from Ref. [24].

**Figure 6:**
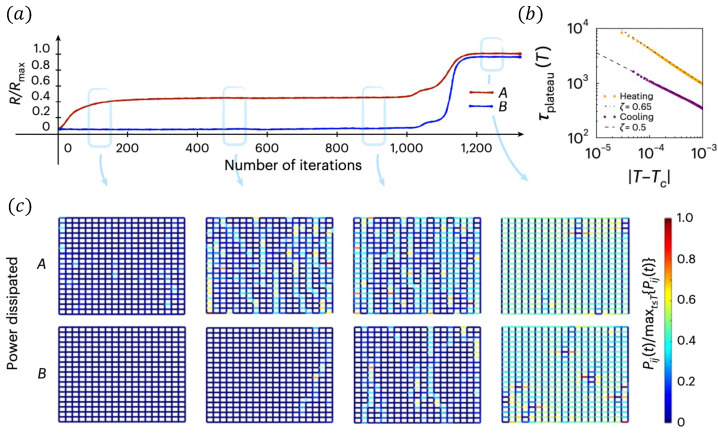
Plateau in interdependent superconducting networks. (**a**) During the abrupt transition at the critical temperature, a plateau is observed. (**b**) The plateau timescale follows the scaling relations $\tau ∼|T-T_{c}{|}^{1/2}$, both from the cooling and heating directions. (**c**) Snapshots of the power dissipated by each segment during the plateau. Figure obtained from Ref. [24].

Interdependent superconducting networks (ISNs) are the first experimentally testable physical interdependent networks that experimentally prove [24] the theory of percolation of interdependent networks [10,16,33]. A single 2D superconducting network of size N=L×L is made of niobium or aluminum dioxide and a biased current $I_{b}$ is induced. The state of each segment (i,j) depends on the potential $V_{ij}$, the current flow $I_{ij}$, its critical current $I_{ij}^{c}$, its critical temperature $T_{ij}^{c}$, and the temperature of the system *T*. Each segment is characterized by Josephson characteristics and can be in one of three states: (SC) superconductor with resistance $R_{SC}\to 0$, (I) intermediate $R_{I}=V_{ij}/I_{ij}$, and (N) normal with $R_{N}=R_{0}$. Theoretically, the global resistance of the network is calculated using the Kirchhoff equation and solved iteratively until a steady state is reached. At low temperatures, all the segments are at the SC-state and the global resistance is zero. As the temperature increases, some segments change their state to I-state or N-state, and at a critical point $T_{c}$, the system resistance percolates and the global resistance increases continuously from zero and a second-order superconducting–normal transition is experimentally observed (see Figure 5a). ISN is made of two thermally coupled 2D superconducting networks (Figure 4). The thermal coupling is a result of ohmic dissipation between the networks. The layer between the networks isolates electricity but conducts heat. At low temperatures, all the segments are at the SC state. When temperature increases, a segment changes its state and starts to dissipate heat. This heat affects some segments at the other network and changes their state, causing them to dissipate heat back to the first network. This can initiate a cascade of heat between the networks, ending when all the segments on both networks are at the N-state and an abrupt transition is observed (Figure 5b). Similar to percolation on interdependent networks and similar to interdependent ferromagnetic networks, also, here, at the critical temperature, a plateau is observed both theoretically and experimentally [25] where the resistance stays almost constant (decays microscopically) for a long time and a single segment in one layer affects on average a single element in the other layer at a given time window (Figure 6a). The plateau timescale follows the same scaling as the ones observed for percolation $\tau ∼|T-T_{c}{|}^{-1/2}$, both from the heating and cooling directions (Figure 6b). By tracking the hotspots of the networks, we can track in simulations how the microscopic changes affect the redistribution of currents (Figure 6c). At the start of the plateau from the heating direction, all the segments are at the SC-state and there is no dissipation. However, during the plateau, some segments start to change their state to the N-state, creating hotspots of heat dissipation. At the end of the plateau, abruptly, all the segments are at the N-state, and there is a uniform dissipation of the entire sample.

## 5. Discussion

Applying the paradigm of abstract interdependent networks in physical systems is crucial to experimentally test and ultimately validate its theoretical predictions. Here, we showed the transition from the abstract percolation of interdependent networks to a more physical model of IFNs and, finally, to the recent experimentally testable setup of ISNs. While percolation is an abstract model and an ISN is an experimentally testable disordered system, the fundamental phenomena are the same. In both systems, spontaneous cascading microscopic changes occur between the networks due to interdependent coupling, which results in an abrupt phase transition with a long macroscopic plateau. The similarity of the phenomena observed in both systems shows the strength of percolation as an abstract paradigm for predicting and discovering novel phenomena in real physical systems. Furthermore, the unique microscopic cascade mechanism observed in both systems is expected to be found in other systems experiencing spontaneous cascading phenomena during their transition [34,35,36,37,38,39,40,41,42,43]. These results and further expected exciting phenomena should encourage researchers to push forward this research on the physical manifestation of interdependent networks down two avenues. The first is by constructing additional physical laboratory setups of interdependent networks in other physical systems, and the IFN is a great example that so far has only been studied theoretically and can be experimentally tested using layers of granular ferromagnets [44,45]. The second path is to further study the abstract percolation paradigm, which can provide a simpler benchmark for discovering novel critical phenomena that can later be experimentally tested and observed in more complex experimental setups. More complex setups include multi-layer systems with more than two layers, which have possible applications for novel multi-layer materials [46,47]. Pursuing both avenues will guarantee the flourishing of the newly open research field of physical manifestation of the interdependent networks’ paradigm.

## Data Availability

The raw data supporting the conclusions of this article will be made available by the authors on request.

## Abbreviations

The following abbreviations are used in this manuscript:

GCC	giant connected component
MGCC	mutual giant connected component
IFN	interdependent ferromagnetic networks
ISN	interdependent superconducting networks
SC-state	superconducting state
N-state	normal state

## References

[1] Bunde A., Havlin S. (2012). Fractals and Disordered Systems.

[2] Stauffer D., Aharony A. (2018). Introduction to Percolation Theory.

[3] Newman M. (2018). Networks.

[4] Albert R., Barabási A.L. (2002). Statistical mechanics of complex networks. Rev. Mod. Phys..

[5] Cohen R., Havlin S. (2010). Complex Networks: Structure, Robustness and Function.

[6] Erdős P., Rényi A. (1959). On Random Graphs I. Publ. Math. Debr..

[7] Erdős P., Rényi A. (1960). On the evolution of random graphs. Publ. Math. Inst. Hung. Acad. Sci.

[8] Bollobás B., Bollobás B. (1998). Random Graphs.

[9] Newman M., Ziff R.M. (2000). Efficient Monte Carlo algorithm and high-precision results for percolation. Phys. Rev. Lett..

[10] Buldyrev S.V., Parshani R., Paul G., Stanley H.E., Havlin S. (2010). Catastrophic cascade of failures in interdependent networks. Nature.

[11] Alert R., Tierno P., Casademunt J. (2017). Mixed-order phase transition in a colloidal crystal. Proc. Natl. Acad. Sci. USA.

[12] Gross B., Bonamassa I., Havlin S. (2022). Fractal fluctuations at mixed-order transitions in interdependent networks. Phys. Rev. Lett..

[13] Gao J., Li D., Havlin S. (2014). From a single network to a network of networks. Natl. Sci. Rev..

[14] Berezin Y., Bashan A., Danziger M.M., Li D., Havlin S. (2015). Localized attacks on spatially embedded networks with dependencies. Sci. Rep..

[15] Parshani R., Buldyrev S.V., Havlin S. (2010). Interdependent networks: Reducing the coupling strength leads to a change from a first to second order percolation transition. Phys. Rev. Lett..

[16] Zhou D., Bashan A., Cohen R., Berezin Y., Shnerb N., Havlin S. (2014). Simultaneous first-and second-order percolation transitions in interdependent networks. Phys. Rev. E.

[17] Dorogovtsev S.N., Goltsev A.V., Mendes J.F.F. (2006). K-core organization of complex networks. Phys. Rev. Lett..

[18] Lee D., Jo M., Kahng B. (2016). Critical behavior of k-core percolation: Numerical studies. Phys. Rev. E.

[19] Gao S., Xue L., Gross B., She Z., Li D., Havlin S. (2024). Possible origin for the similar phase transitions in k-core and interdependent networks. New J. Phys..

[20] Xue L., Gao S., Gallos L.K., Levy O., Gross B., Di Z., Havlin S. (2024). Nucleation phenomena and extreme vulnerability of spatial k-core systems. Nat. Commun..

[21] Motter A.E., Lai Y.C. (2002). Cascade-based attacks on complex networks. Phys. Rev. E.

[22] Motter A.E. (2004). Cascade control and defense in complex networks. Phys. Rev. Lett..

[23] Perez I.A., Ben Porath D., La Rocca C.E., Braunstein L.A., Havlin S. (2024). Critical behavior of cascading failures in overloaded networks. Phys. Rev. E.

[24] Bonamassa I., Gross B., Laav M., Volotsenko I., Frydman A., Havlin S. (2023). Interdependent superconducting networks. Nat. Phys..

[25] Gross B., Volotsenko I., Bonamassa I., Havlin S., Frydman A. (2024). Microscopic origin of abrupt transition in interdependent superconducting networks. arXiv.

[26] Danziger M.M., Bashan A., Havlin S. (2015). Interdependent resistor networks with process-based dependency. New J. Phys..

[27] Bonamassa I., Gross B., Havlin S. (2021). Interdependent couplings map to thermal, higher-order interactions. arXiv.

[28] Gross B., Bonamassa I., Havlin S. (2024). Microscopic Intervention Yields Abrupt Transition in Interdependent Ferromagnetic Networks. Phys. Rev. Lett..

[29] Ising E. (1924). Beitrag zur Theorie des Ferro-und Paramagnetismus. Ph.D. Thesis.

[30] McCoy B.M., Wu T.T. (1973). The Two-Dimensional Ising Model.

[31] Pippard A.B. (1989). Magnetoresistance in Metals.

[32] Xiao J.Q., Jiang J.S., Chien C.L. (1992). Giant magnetoresistance in nonmultilayer magnetic systems. Phys. Rev. Lett..

[33] Gao J., Buldyrev S.V., Stanley H.E., Havlin S. (2012). Networks formed from interdependent networks. Nat. Phys..

[34] Zapperi S., Vespignani A., Stanley H.E. (1997). Plasticity and avalanche behaviour in microfracturing phenomena. Nature.

[35] Zapperi S., Lauritsen K.B., Stanley H.E. (1995). Self-organized branching processes: Mean-field theory for avalanches. Phys. Rev. Lett..

[36] Alava M.J., Nukala P.K., Zapperi S. (2006). Statistical models of fracture. Adv. Phys..

[37] Pradhan S., Hansen A., Chakrabarti B.K. (2010). Failure processes in elastic fiber bundles. Rev. Mod. Phys..

[38] Rundle J.B., Turcotte D.L., Shcherbakov R., Klein W., Sammis C. (2003). Statistical physics approach to understanding the multiscale dynamics of earthquake fault systems. Rev. Geophys..

[39] Peng H., Zhao Y., Zhao D., Zhong M., Hu Z., Han J., Li R., Wang W. (2023). Robustness of higher-order interdependent networks. Chaos Solitons Fractals.

[40] Lai Y., Liu Y., Zheng K., Wang W. (2023). Robustness of interdependent higher-order networks. Chaos Interdiscip. J. Nonlinear Sci..

[41] Qian C., Zhao D., Zhong M., Peng H., Wang W. (2024). Cascading failures on interdependent hypergraph. Commun. Nonlinear Sci. Numer. Simul..

[42] Chen L., Zhu Y., Meng F., Liu R.R. (2024). Catastrophic cascade of failures in interdependent hypergraphs. Chaos Interdiscip. J. Nonlinear Sci..

[43] Liu R.R., Chu C., Meng F. (2023). Higher-order interdependent percolation on hypergraphs. Chaos Solitons Fractals.

[44] Gerber A., Milner A., Groisman B., Karpovsky M., Gladkikh A., Sulpice A. (1997). Magnetoresistance of granular ferromagnets. Phys. Rev. B.

[45] Milner A., Gerber A., Groisman B., Karpovsky M., Gladkikh A. (1996). Spin-dependent electronic transport in granular ferromagnets. Phys. Rev. Lett..

[46] Nika G., Constantinescu A. (2019). Design of multi-layer materials using inverse homogenization and a level set method. Comput. Methods Appl. Mech. Eng..

[47] Roudgé M., Cherif M., Cahuc O., Darnis P., Danis M. (2008). Multi-layer materials. Qualitative approach of the process. Int. J. Mater. Form..

